# Post-surgical outcomes in transgender women: a prospective analysis of sexual function and health-related quality of life

**DOI:** 10.1007/s00345-025-05887-9

**Published:** 2025-09-02

**Authors:** M. J. Wenk, N. Rademacher, B. Liedl, B. Grüne, B. Meister

**Affiliations:** 1 Department of Urology and Urological Surgery, University Medical Center Mannheim, University of Heidelberg, Theodor-Kutzer-Ufer 1-3, 68167 Mannheim, Germany; 2 Center of Reconstructive Urogenital Surgery, Urologische Klinik Planegg, Planegg, Germany

**Keywords:** Gender affirming surgery, Gender dysphoria, Transgender women, Sexual function, Quality of life

## Abstract

**Purpose:**

To prospectively evaluate patient and clinician reported outcomes of sexual function and health-related quality of life (QOL) of transgender women preoperatively (t0), 6 months (t1), and 12 months (t2) after gender-affirming surgery (GAS) with penile inversion vaginoplasty.

**Methods:**

Transgender women undergoing two-stage GAS at our tertiary care center were included (04/2019–01/2023). Patients received a composed questionnaire including a validated questionnaire (SF-12). Clinical outcomes and adverse events were evaluated. A regression analysis was performed to identify possible risk factors for a deterioration in sexual function and QOL.

**Results:**

Fifty-three patients with an average age of 40.27 ± 14.43 years were included. Complications were mostly minor (Clavien Dindo Classification grade I/II). The depth of the neovagina postoperatively was 11.98 ± 2.19 cm and the width 3.44 ± 0.98 cm. 79.4% of patients were able to experience an orgasm 12 months postoperatively. Orgasm quality increased significantly over the measurement time points (*p* = 0.003). The SF-12 average physical and mental scores of our collective were similar to the German populations average scores with no significant differences between the time measurement points (*p* = 0.405 and *p* = 0.198, respectively). The mental score was always lower than the physical score. A multiple linear regression analysis showed age < 40 years to be a significant influence factor regarding the physical QOL-score (*p* = 0.048) and the ability to experience an orgasm (*p* = 0.026).

**Conclusion:**

Penile inversion vaginoplasty is a safe procedure that yields favorable outcomes regarding sexual function and health-related QOL. Younger age significantly predicts improved physical QOL and the ability to experience orgasm postoperatively.

**Supplementary Information:**

The online version contains supplementary material available at 10.1007/s00345-025-05887-9.

## Introduction

The term “transgender” refers to individuals whose gender identity does not align with the sex assigned to them at birth based on physical characteristics [1]. This incongruence often leads to significant distress, commonly referred to as gender dysphoria (GD) [1]. Transgender individuals frequently face a range of challenges, including physiological issues, social obstacles such as lack of support, discrimination, victimization, rejection, and transphobia, as well as psychological difficulties such as anxiety, depression, and low self-esteem [2–4]. These factors can negatively impact both quality of life (QOL) and health perception [5].

Gender-affirming treatment aims to align an individual’s physical characteristics with their gender identity, which can effectively alleviate GD and improve health-related QOL [1].

Many individuals seek gender-affirming surgery (GAS), a highly specialized and complex surgical discipline. For transgender women, this typically involves vulvo-vaginoplasty, which may include excision of scrotal skin, orchiectomy, urethroplasty, labioplasty, clitoroplasty, recto-vesico-prostatic dissection, and creation of a vaginal cavity [6, 7]. The primary goal is to construct a perineogenital complex that closely resembles female anatomy in both appearance and function, providing a sensitive clitoris for orgasm and a vagina suitable for receptive intercourse [8].

Although recent reviews suggest promising outcomes regarding satisfaction and QOL following GAS, most studies are limited by small sample sizes and observational designs, preventing causal conclusions [9]. Sexual health is an important component of QOL but remains difficult to assess due to its multifaceted nature [10]. Notably, a study by Barcelos et al. reported only weak evidence that GAS improves sexual function in transgender women [11]. Furthermore, most existing studies on sexual function after GAS rely on cross-sectional or cohort designs [10], and prospective data are scarce.

The aim of our study was to prospectively evaluate patient-reported outcome measures (PROMs) on sexual function and health-related QOL in transgender women before surgery and at 6 and 12 months postoperatively, following penile inversion vaginoplasty. Additionally, we report clinician-reported outcome measures (CROMs) related to surgical results. This study aims to provide more robust data on surgical outcomes, QOL, and sexual function, and to help identify key factors influencing successful outcomes.

## Patients and methods

### Study population and data collection

After institutional ethics review board (Ethics committee II Mannheim, University of Heidelberg) approval was received, patient data were prospectively collected upon patient consent (informed consent was obtained from all individual participants included in the study). The study was conducted in accordance with the principles of the Declaration of Helsinki. All transgender women who underwent GAS in our hospital from 04/2019 until 01/2023, who agreed to participate in the study and were able to complete the questionnaire in German, were included.

### Surgical technique and examination

In all cases, vulvo-vaginoplasty was carried out by removing the scrotal skin, performing orchiectomy, urethroplasty, labioplasty, clitoroplasty, dissecting the recto-vesico-prostatic plane, and creating a vaginal cavity through penile skin inversion combined with a skin scrotal graft. Following this primary procedure, patients returned for a scheduled second-stage surgery approximately six months later. During this follow-up operation, the remaining perineal skin was opened, and cosmetic or functional refinements were made as needed. Intraoperatively, assessments were conducted to evaluate the length and width of the neovagina, as well as to determine the need for corrective measures in cases of stenosis (CROMs).

### Questionnaires

All included patients completed a validated questionnaire assessing general health perception: The 12-item Short Form (SF-12) Survey. The questionnaire has been shown to be valid and reliable in German translation [12]. Two summary scores are reported: a mental component score (MCS-12) and a physical component score (PCS-12). The scores may be reported as Z-scores (difference compared to the population average, measured in standard deviations). The German population average PCS-12 is 49.6 *±* 8.7 points and MCS-12 52.3 *±* 8.0 points [13].

Additionally, a self-administered, non-validated questionnaire on demographic, general, and health care data, as well as other symptoms (14 questions, various symptoms including voiding, pain, general health perception, satisfaction, and sexual function) was completed by each patient. At the time this study was designed, no validated German questionnaires specifically addressing sexual function in transgender individuals were available. Due to the large amount of data, only the PROMS related to sexual function and general health perception of the self-administered questionnaire are presented here. Specifically, some questions addressed specific domains. Those were: sexual activity (behavior), vaginal intercourse (behavior), sexual orientation, ability to experience an orgasm (orgasm), sensation in the neoclitoris (arousal), sensation in the neovagina (arousal), orgasm quality (orgasm), sexually stimulating feeling in the neoclitoris (arousal), pain in the neoclitoris (pain), sexually stimulating feeling in the neovagina (arousal), and pain in the neovagina (pain).

### Clinical parameters

The following preoperative parameters were prospectively collected from the patients’ electronic medical records: Age, body mass index (BMI), and the American Society of Anesthesiologists (ASA) score. In addition, we recorded the following perioperative parameters: operation time, blood loss, state after circumcision, length of the glans, length of the clitoris, length of the skin mesh, and length and width of the neovagina. For the purpose of geometric comparison, the glans and the clitoris were approximated as circular structures. The maximal diameter of each structure was measured intraoperatively using a sterile ruler. Neovaginal depth was measured intraoperatively using a Kristeller vaginal speculum with a standardized length of 11 cm. In cases where the neovaginal depth exceeded this length, a sterile measuring tape was used to determine the total depth. Vaginal width was assessed manually by digital examination and confirmed using a sterile measuring tape. Furthermore, reinterventions and complications according to the Clavien-Dindo classification (CDC) as well as the Complication Comprehensive Index (CCI) [14] until the first discharge were evaluated.

### Statistical analysis

Descriptive statistics were carried out to present the baseline and perioperative characteristics. Quantitative data were expressed as mean and standard deviation (SD), and categorical data as absolute and relative frequencies. All continuous variables were examined for normal distribution using the Shapiro-Wilk test and were found to be non-normally distributed (p-value < 0.05 in each case). Due to the non-normal distribution of the data, a data transformation was carried out using logarithmization. The comparison of the variables at the three measurement times was carried out after the corresponding logarithmization with an analysis of variance repeated measure. In case of significant differences between the three measurement times t0, t1, and t2, a post-hoc test with Bonferroni correction was performed to determine where exactly the significant difference lay.

For the comparison of ordinal scaled variables over time, a Generalized Estimating Equations model was used. In the analyses over time, only patients with reported data at all measurements points were suitable for analyses, e.g. at 12 months, 29 patients were included in the longitudinal SF-12 analysis. The remaining 24 participants were excluded due to incomplete responses—specifically, missing individual items within the SF-12 that prevented the calculation of valid PCS and MCS summary scores. These participants were not lost to follow-up in general and often completed other parts of the questionnaire. Therefore, they were excluded from the longitudinal analysis, but no formal dropout analysis was conducted.

Predictive factors for better postoperative sexual function were identified using a stepwise multiple linear regression analysis. All statistical analyses were carried out with Jump version 14 (Statistical Analysis System Institute, Cary, United States of America). The level of significance was set at 0.05.

## Results

### Baseline characteristics

A total of 53 transgender women who underwent GAS at our department between April 2019 und January 2023 were included in the analysis. Baseline characteristics of the patient cohort are summarized in Table 1. 47 patients underwent the second GAS. Mean age was 40.27 ± 14.43 years, mean BMI was slightly overweight with 25.76 ± 6.05 kg/m², mean operation time was 289.16 ± 60.23 min, and mean blood loss about half a liter (585.71 ± 254.13 ml).

During the first operation, the length of the glans was 3.34 *±* 0.56 cm, which reduced to 2.28 *±* 0.62 cm after shaping the neoclitoris. During the second operation the size of the neovagina was measured at 11.98 *±* 2.19 cm (depth) and 3.44 *±* 0.98 (width). The length of the neoclitoris was 2.29 *±* 0.69 cm.

Complications were reported in nine patients after the first surgery, with most of them being minor CDC grades I or II. Two patients (3.77%) suffered CDC *≥* III complications (one rectal perforation, one patient needed a suprapubic catheter postoperatively). After the second operation, one patient (2.56%) had a complication in the form of bleeding that required hemostasis in general anesthesia. In five patients, an incision of scar tissue was performed to address a relative neovaginal stenosis. Two patients required surgical intervention for the correction of a true neovaginal stenosis.

**Table 1:** Baseline characteristics and perioperative parameters

Variable	n = 53
Age [years] (Mean ± SD)	40.27 ± 14.43
BMI [kg/m²] (Mean ± SD)	25.76 ± 6.05
ASA-Score (Median, IQR)	2.00 (1.00–2.00)
Blood loss [ml] (Mean ± SD)	585.71 ± 254.13
Operation time [min] (Mean ± SD)	289.16 ± 60.23
State after circumcision (n, %)	10 (18.87)

Intraoperative measures first operation	n = 50
Length glans [cm] (Mean ± SD)	3.34 ± 0.56
Length clitoris [cm] (Mean ± SD)	2.28 ± 0.62
Length skin mesh [cm] (Mean ± SD)	5.84 ± 3.07

Intraoperative measures second operation	n = 47
Depth of Neovagina [cm] (Mean ± SD)	11.98 ± 2.19
Width of Neovagina [cm] (Mean ± SD)	3.44 ± 0.98
Length clitoris [cm] (Mean ± SD)	2.29 ± 0.69

Complications first operation	n = 53
Clavien Dindo I (n, %)	2 (3.77)
Clavien Dindo II (n, %)	2 (3.77)
Clavien Dindo III a (n, %)	1 (1.89)
Clavien Dindo IV a (n, %)	1 (1.89)
Comprehensive complication index (Mean ± SD)	18.26 ± 9.91

Complications second operation	n = 47
Clavien Dindo III b (n, %)	1 (2.13)
Comprehensive complication index	33.70

*BMI* body mass index; *SD* standard deviation; *n* number

Sexual function over time is shown in Table 2. Patients were asked about their sexual activity, sexual orientation and if they had vaginal intercourse. They were also asked about their ability to have an orgasm, and if sensation in the clitoris and neovagina occurred. Additionally, the orgasm quality, pain in the clitoris or neovagina, the ability to experience a sexually stimulating feeling in the clitoris and neovagina, as well as the sensation of the skin in the neovagina were reported. The responses were evaluated preoperatively (t0), 6 months (t1) and 12 months (t2) after surgery. There were no significant changes for any of the parameters between the time measurement points except for two parameters: vaginal intercourse, which increased significantly from t1 to t2 (*p* = 0.014), and the orgasm quality. Orgasm quality increased significantly over the measurement time points (*p* = 0.003), and the post-hoc test showed that the significant differences were between t0 and t1 (*p* = 0.013), as well as t0 and t2 (*p* = 0.004). No pain at all (answer 0 on the scale from 0 to 10) in the neoclitoris was reported by 7 patients (21.9%) at t1 and 9 patients at t2 (28.1%). No pain at all (answer 0 on the scale from 0 to 10) in the neovagina was reported by 9 patients (29.0%) at t1 and 12 patients at t2 (38.7%).

**Table 2:** Sexual function over time

Variable		n = 53			p
(%)		t0	t1	t2	
Sexual activity	Yes	27 (54.00)	33 (64.70)	25 (73.50)	0.053
	No	23 (46.00)	18 (35.30)	9 (26.50)	
Vaginal intercourse	Yes	–	9 (18.40)	8 (24.20)	**0.014**
	No	–	40 (81.60)	25 (75.80)	
Sexual orientation	Female	17 (34.00)	16 (31.40)	11 (32.40)	0.606
	Male	11 (22.00)	12 (23.50)	7 (20.60)	
	Both	15 (30.00)	20 (39.20)	9 (26.50)	
	Non-binary	7 (14.00)	3 (5.90)	7 (20.60)	
Ability to experience an orgasm	Yes	42 (84.00)	37 (74.00)	27 (79.40)	0.458
	No	8 (16.00)	13 (26.00)	7 (20.60)	
Sensation in the neoclitoris	Completely	–	24 (47.10)	18 (52.90)	0.144
	Mostly		14 (27.50)	9 (26.50)	
	A little bit		11 (21.60)	6 (17.60)	
	No		2 (3.90)	1 (2.90)	
Sensation in the neovagina	Completely	–	9 (18.40)	11 (33.30)	0.423
	Mostly		21 (42.90)	11 (33.30)	
	A little bit		15 (30.60)	7 (21.20)	
	No		4 (8.20)	4 (12.10)	

Mean (SD)	t0	t1	t2	*p*	Post hoc *p*
Orgasm quality^a^ (n = 27)	4.15 ± 3.09	6.15 ± 3.23	6.63 ± 3.30	**0.003**	t0 vs. t1 p = *0.013* t1 vs. t2 *p* = 0.117 t0 vs. t2 p = *0.004*
Sexually stimulating feeling in the neoclitoris^a^ (n = 32)	-	6.34 ± 3.05	6.84 ± 3.18	0.151	
Pain in the neoclitoris^c^ (n = 32)	-	2.56 ± 2.15	2.59 ± 2.39	0.980	
Sexually stimulating feeling in the neovagina^b^ (n = 30)	-	5.13 ± 3.31	5.40 ± 3.67	0.404	
Touching of the skin in the neovagina^b^ (n = 31)	-	5.97 ± 2.85	6.13 ± 2.84	0.752	
Pain in the neovagina^c^ (n = 31)	-	1.94 ± 1.81	1.99 ± 1.84	0.939	

[a] 0—not at all satisfying 10—completely satisfying; [b] 0—no not at all 10—yes, completely; [c] 0—no pain 10—extreme painn = number; SD = standard deviation

Table 3 shows the results of the SF-12 questionnaire. The MCS- and PCS-scores were relatively high at all measurements points, showing good general health. The MCS Z-scores were all below the scores of the German average population, whereas the PCS Z-scores were all higher than the average. There were no significant differences in the PCS- or MCS-scores between the measurement time points (*p* = 0.405 and *p* = 0.198, respectively), so no significant change in the general state of health and health related QOL could be determined.

**Table 3:** SF-12 questionnaire over time

SF-12 Variable	*n* = 29			*p*
Mean (SD)	t0	t1	t2	
PCS score	54.11 ± 7.90	51.90 ± 7.11	52.82 ± 8.79	0.405
MCS score	42.19 ± 13.19	47.46 ± 11.96	45.05 ± 13.05	0.198
PCS Z score	4.51	2.30	3.22	
MCS Z score	− 10.11	− 4.84	− 7.25	

*SF-12* 12-item Short Form Survey; *n* number; *PCS* physical component score; *MCS* mental component score; *Z-score* difference compared to population average

A multiple linear regression analysis was performed to evaluate possible factors influencing the general health perception. For the PCS- and MCS-score, age, pain intensity and satisfaction with the cosmetical and functional result were tested. Linear regression showed only age < 40 years to be a significant influence factor for PCS (< 40 vs. >40 years of age, odds ratio (OR) 5.263, 95% confidence interval (CI) 1.018–27.211, *p* = 0.048). For the MCS score, none of the factors showed a significant influence in the regression analysis.

Regarding the ability to experience an orgasm, additionally to the above-mentioned parameters the state after circumcision, the length of the neoclitoris as well as the depth of the neovagina were tested. Again, age < 40 years was the only significant influence factor (< 40 vs. >40 years of age, OR 4.687, CI 1.198–18.340, *p* = 0.026).

## Discussion

This study prospectively evaluated sexual function and health-related quality of life (QOL) in transgender women before, 6 months, and 12 months after penile inversion vaginoplasty. Postoperative complications were predominantly minor. One year post-surgery, most patients reported the ability to achieve orgasm, with a significant improvement in orgasm quality over time. Health-related QOL scores were comparable to those of the general German population; however, mental well-being consistently scored lower than physical health, underscoring the need for ongoing care and support beyond surgery.

A persistent challenge in this field is the lack of standardized reporting on functional outcomes, due to variations in surgical techniques, specialties, and inconsistent terminology over time [10, 15]. While recent studies have aimed to address this using validated tools, most were developed for cisgender populations [16]. When we designed this study in 2018, no validated questionnaires specific to transgender individuals were available. Although tools like the AFFIRM and oMtFSFI questionnaires have since been developed [17, 18], they were not yet validated in German, and changing instruments mid-study would have compromised our data.

### Sexual function

#### Anatomical aspects and sexual activity

A systematic review with meta-analysis found that the average, quantitatively measured neovaginal depth after GAS was 12.2 cm (range 6.5–16.5 cm, 57 studies, 2944 patients) and the average vaginal width was 3.2 cm (range 2.8–3.9 cm, 17 studies, 823 patients) [15]. This aligns with the measurements of the average 11.9 cm for neovaginal length and 3.4 cm width we found in our study. Sexual activity rates one year post-surgery (73.5%) mirrored those in previous studies (73.7%).

However, measurement techniques for vaginal depth vary across studies, complicating comparisons and underscoring the need for standardized protocols.

#### Sensibility

Preserving sensibility is a key objective in GAS, requiring meticulous neurovascular preparation. Ramsay et al. found sensibility in the neoclitoris in 96.6% and 90.0% at 6 and 12 months, respectively [16]; our data similarly showed 96.1% and 97.1%. Pain after GAS is common, ranging from surgical trauma to hypersensitivity, particularly of the clitoris. Ramsay et al. reported 75.9% of patients had pain in or around the neovagina after 6 months, and 56.8% after 12 months; we observed comparable rates (71.0% at 6 months; 61.3% at 12 months). However, reported pain levels were low (mean < 2/10 at both time points) and largely not bothersome, similar to findings from Zavlin et al. (mean 2.33/10).

Goddard et al. reported 14% of their patients to experience “uncomfortable sensation” due to hypersensitivity at the neoclitoris [17]. We found pain of any kind (> 0/10) in the neoclitoris in 78.1% at t1 and 71.9% at t2, which is markable higher than in Goddards collective. The reason needs to be investigated in our future research, even though, similar to pain in the neovagina, the bother intensity was relatively low (< 3/10) at both t1 and t2.

#### Orgasm

A systematic review found that the overall percentages of transgender women after GAS who were able to achieve an orgasm ranged between 17.4 and 100% (median 79.7%, *n* = 2384) [10]. Another systematic review of 57 studies found that 76% of transwomen were able to achieve an orgasm after vaginoplasty [18]. This is in accordance with our results (the ability to experience an orgasm was 74.0% after 6 months and 79.4% after 12 months). In general, not being able to achieve an orgasm is one of the most common problems after GAS. However, orgasmic function is multifactorial, influenced not only by surgical outcomes but also by factors such as medications, mental health, and pre-existing neurological conditions. Therefore it is crucial to assess the status preoperatively and to counsel patients accordingly to set realistic expectations (a preexisting anorgasmia might not be cured by GAS).

### General health perception QOL

QOL results in the literature are ambiguous. Transgender individuals are known to be at high risk of developing mental health problems [19], which is understandable given the many obstacles they endure on their transition journey. The statement is also supported by the following studies, which all show lower average MCS- than PCS-scores.

A Chinese study by Yang et al. reported average PCS-scores of 82.4 and MCS-scores of 68.1 for transgender women using the SF long form (SF-36), which is comparable to the SF-12 [20]. This seems high compared to the scores found in our study (around 51.9–54.1 for the PCS-score and 42.2–47.5 for the MCS-score, depending on the time measurement point), and also very high given the fact that the Chinese population SF-36 average for both the PCS- and the MCS-score is 50.0 points [21], which is comparable to the German population average scores (PCS-12 49.6 points and MCS-12 52.3 points) [13]. Our scores are in accordance with other studies such as a study in a Spanish transgender women collective (SF-36 mean PCS-score 54.37 and mean MCS-score 48.63) [22] and a United States transgender women collective (mean PCS-score 53.5 and mean MCS-score 49.3) [23] after GAS. An Iranian study on the contrary showed low average PCS- (39.2) und MCS- (40.2) scores after GAS (and even lower before). It must be said that all studies were performed with a small sample size.

These comparisons highlight the influence of broader life circumstances, cultural factors, and healthcare systems on QOL. Notably and like in all other studies, MCS scores remained below the general population average at all time points, despite postoperative improvements. This indicates that while GAS may enhance mental well-being, it does not resolve underlying psychosocial stressors such as stigma and minority stress. These findings highlight the need for comprehensive care that includes mental health support alongside surgery.

Our regression analysis identified age < 40 as a significant factor for better PCS scores, supporting data validity. Other predictors were not identified, likely due to limited sample size, but warrant exploration in future, larger studies.

It is important to acknowledge that 24 participants were excluded from the longitudinal SF-12 analysis due to incomplete questionnaire responses. Unfortunately, the potential for response bias remains—particularly if individuals experiencing poorer mental health were more likely to leave items unanswered. This may have led to an overestimation of mental health-related QOL in the analyzed sample.

Additionally, the timing of the second-stage surgery may have influenced outcome interpretation. This procedure was performed no earlier than six months after the initial surgery. The 6-month follow-up was always performed beforehand, reflecting only the effects of the primary surgery. The 12-month follow-up was performed after the second stage, though the timing varied between patients. As a result, differences in the interval between surgery and follow-up may have affected individual outcomes, particularly for sexual function and vaginal depth. This variability should be considered when interpreting the results.

Limitations:

Due to the Covid pandemic, fewer patients than initially planned received GAS during the study period. Additionally, the response rate for fully completed questionnaires across all time points was lower than anticipated, with the SF-12 reaching 54.72%. Health-related QOL was assessed using the SF-12 questionnaire which has not been specifically validated for transgender, and sexual function was assessed with open questions and no validated tool.

Nevertheless, patient-centered studies with PROMS are essential for improving patient care. It facilitates informed, patient-centered counseling and supports the establishment of realistic expectations regarding both functional and aesthetic outcomes. This contributes significantly to postoperative satisfaction, psychosocial well-being, and long-term adherence to care. The prospective design of our study is a significant strength, and we hope it encourages further research to assess surgical outcomes more objectively, ideally using specifically validated instruments.

## Conclusion

GAS with penile inversion vaginoplasty is a safe procedure that results in a significant improvement in orgasm quality for transgender women. There were no significant changes in health related QOL. Younger age < 40 years appears to be a predictor of better outcomes in both physical QOL and sexual function. While the overall findings are promising, continued research and long-term follow-up are needed to further optimize patient care and counseling, and to set realistic expectations for both functional and aesthetic outcomes. Validated tools for this specific patient population are desperately needed.

## Supplementary Information

Below is the link to the electronic supplementary material.

Supplementary Material 1

Supplementary Material 2

## Data Availability

No datasets were generated or analysed during the current study.
