# Supplementary material for: Post-surgical outcomes in transgender women: a prospective analysis of sexual function and health-related quality of life
Source: World J Urol. 2025 Sep 2;43(1):529. doi: 10.1007/s00345-025-05887-9 (PMC12405499; doi:10.1007/s00345-025-05887-9)
Supplement: Supplementary file 1 — Supplementary Material 1 [file 345_2025_5887_MOESM1_ESM.pdf]

Medizinische Fakultät Mannheim
der Universität Heidelberg
Universitätsklinikum Mannheim

Universitätsmedizin Mannheim
Klinik für Urologie und Urochirurgie
Direktor: Prof. Dr. med. M. S. Michel

FO Erhebungsbogen GD MzF – Korrekturen

Name:
Vorname:
Geb.:
oder Etikett

Erhebungsdatum: ____________________________

Telefonnummer: ____________________________

E-Mail Adresse: ____________________________

Sehr geehrte Patientin,

es ist unser Anliegen, unsere Behandlung so gut wie möglich an Ihre Beschwerden sowie an Ihre Bedürfnisse und Erwartungen anzupassen.

Deshalb bitten wir Sie, die folgenden Fragen möglichst vollständig zu beantworten. Hiermit helfen Sie uns, die Ergebnisse der bisherigen Operationen zu erfassen, um so die Qualität unserer Arbeit beurteilen und stetig verbessern zu können.

Selbstverständlich erfolgen Erfassung, Speicherung und Auswertungen Ihrer Daten unter ärztlicher Schweigepflicht und streng wissenschaftlichen Kriterien sowie unter Einhaltung der vorgesehenen Datenschutzvoraussetzungen und Richtlinien.

1. Sind bisher schon geschlechtsangleichende oder andere Operationen im Genitalbereich durchgeführt worden (z.B. Beschneidung)

□ ja □ nein

wenn ja: was? wann? wo? (z.B. Brustaufbau, November 2012, Klinikum xxx)

1. ____
2. ____
3. ____
4. ____
5. ____

FO Erhebungsbogen GD MzF – Korrekturen

2a. Berufliche Situation

□ in Ausbildung □ berufstätig □ arbeitssuchend □ Frührentnerin □ Rentnerin

2b. Schulabschluss

□ keiner □ Volks-/Hauptschule □ Realschule □ Gymnasium □ andere

2c. Partnerschaftsstatus (Mehrfachnennung möglich)

□ feste Partnerschaft □ verheiratet □ verwitwet □ keine Partnerschaft □ wechselnde Sexualpartner

3. Rauchgewohnheiten:

□ Raucherin seit ____ Jahren; ____ Zigaretten/Tag

□ Ex-Raucherin seit ____ Jahren

□ Nichtraucherin

4a. Sind Sie sexuell aktiv? (Geschlechtsverkehr, Selbstbefriedigung oder Ähnliches)

□ ja □ nein

4b. Hatten Sie bereits Geschlechtsverkehr durch Penetration Ihrer Neovagina?

□ ja □ nein

4b. Wie ist Ihre sexuelle Orientierung?

□ Frauen □ Männer □ beide □ divers/ohne Definition

5a. Ist Orgasmusfähigkeit vorhanden?

□ ja □ nein

5b. Wie sehr sind Sie in Bezug auf Ihren Orgasmus zufrieden?

(bitte markieren Sie eine Zahl zwischen 0 [gar nicht] und 10 [voll zufrieden])

(gar nicht) **0** 1 2 3 4 5 6 7 8 9 **10** (voll zufrieden)

Universitätsmedizin Mannheim
Klinik für Urologie und Urochirurgie
Direktor: Prof. Dr. med. M. S. Michel

FO Erhebungsbogen GD MzF – Korrekturen

6a. Leiden Sie unter häufig wiederkehrenden Harnwegsinfekten?

□ ja □ nein

wenn ja wie häufig? □ 1-3 /Jahr □ 3-6 / Jahr □ mehr als 7 / Jahr

6b. Tritt bei diesen Infekten Fieber auf?

□ ja □ nein

7a. Bestehen Schmerzen im Genital- oder Beckenbereich?

□ nie □ selten □ manchmal □ meistens □ immer

7b. Wo treten diese Schmerzen auf (mehrfache Nennungen möglich)?

□ Schamhügel □ Klitoris □ Schamlippen □ Scheideneingang □ Damm □ Scheidenhöhle

7c. Falls ja, wie stark sind die Schmerzen im Genital – oder Beckenbereich?

Bitte markieren Sie eine Zahl zwischen 0 (keine Schmerzen) und 10 (stärkste Schmerzen).

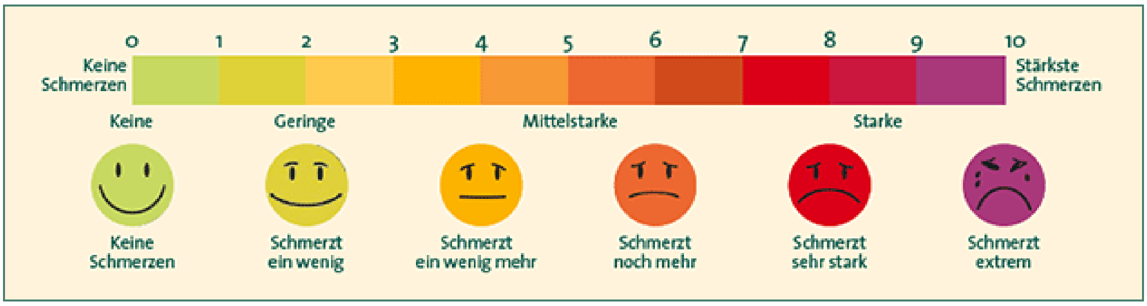

7d. Wie lange bestehen diese Schmerzen schon?

□ kein Schmerz □ kürzer als 3 Monate □ 3-6 Monate □ länger als 6 Monate

8. Empfindlichkeit im Genitalbereich:

8a. Ist die Neoklitoris empfindlich?

□ voll □ zum größten Teil □ etwas □ nein

Wie würden Sie die Art des Gefühls an der Neoklitoris beschreiben?
(bitte je eine Zahl zwischen 0 [trifft gar nicht zu] und 10 [trifft voll zu] markieren)

8b. sexuell erregende Empfindung (bei sexueller Aktivität oder Erregung):

(trifft gar nicht zu) **0** 1 2 3 4 5 6 7 8 9 **10** (trifft voll zu)

8c. Schmerz / unangenehme Empfindung:

(trifft gar nicht zu) **0** 1 2 3 4 5 6 7 8 9 **10** (trifft voll zu)

8d. Ist die Neovagina empfindlich?

□ voll □ zum größten Teil □ etwas □ nein

Wie würden Sie die Art des Gefühls an der Neovagina beschreiben?
(bitte je eine Zahl zwischen 0 [trifft gar nicht zu] und 10 [trifft voll zu] markieren)

8e. sexuell erregende Empfindung (bei sexueller Aktivität oder Erregung):

(trifft gar nicht zu) **0** 1 2 3 4 5 6 7 8 9 **10** (trifft voll zu)

8f. normale Hautberührung:

(trifft gar nicht zu) **0** 1 2 3 4 5 6 7 8 9 **10** (trifft voll zu)

8g. Schmerz / unangenehme Empfindung:

(trifft gar nicht zu) **0** 1 2 3 4 5 6 7 8 9 **10** (trifft voll zu)

9a. Wie sehr sind Sie mit dem bisherigen funktionellen Ergebnis der geschlechtsangleichenden Operationen zufrieden? (bitte markieren Sie eine Zahl zwischen 0 [gar nicht] und 10 [voll zufrieden])

(gar nicht) **0** 1 2 3 4 5 6 7 8 9 **10** (voll zufrieden)

9b. Wie sehr sind Sie mit dem bisherigen kosmetischen Ergebnis der geschlechtsangleichenden Operationen zufrieden? (bitte markieren Sie eine Zahl zwischen 0 [gar nicht] und 10 [voll zufrieden])

(gar nicht) **0** 1 2 3 4 5 6 7 8 9 **10** (voll zufrieden)

10. Bis zu welcher Bougierungsgröße/Dilatorgröße können Sie Ihre Neovagina bougieren?

z.B. Amielle comfort ®

□ Größe 1 (7x1,5cm) □ Größe 2 (9x2cm) □ Größe 3 (11x2,5cm)

□ Größe 4 (14x3cm) □ Größe 5 (16x3,5cm)

z.B. Vagiwell ®

□ Größe 1 (12x1,4cm) □ Größe 2 (13,5x1,8cm) □ Größe 3 (15x2,2cm)

□ Größe 4 (16,3x2,6cm) □ Größe 5 (17,7x3,0cm)

11a. Leiden unter Ausfluss aus der Neovagina?

□ nie □ einmal pro Woche □ mehrmals pro Woche (aber nicht täglich)

□ täglich einmal □ täglich mehrmals

11b. Fühlen Sie sich durch den Ausfluss gestört?

□ gar nicht □ etwas □ sehr □ extrem

Universitätsmedizin Mannheim
Klinik für Urologie und Urochirurgie
Direktor: Prof. Dr. med. M. S. Michel

FO Erhebungsbogen GD MzF – Korrekturen

12. Wie oft führen Sie eine Vaginaldusche durch?

□ nie □ einmal pro Woche □ mehrmals pro Woche (aber nicht täglich)

□ täglich einmal □ täglich mehrmals

13. Verwenden Sie Salben im Bereich Ihrer Neovagina? Falls ja,

□ Östrogensalbe □ Antibiotische Salbe (z.B. Furacin ®) □ Sonstige: ________________

14a. Würden Sie die genitalangleichende Operation nochmals durchführen lassen?
□ ja □ nein

14b. Würden Sie die genitalangleichende Operation einer anderen Person mit Mann zu Frau Genderdysphorie empfehlen?

□ ja □ nein

Es folgen **2 weitere Fragebögen** zu urologischen Beschwerden (ICIQ-MLUTS) und zum allgemeinen Gesundheitszustand (SF12).Bitte beantworten Sie auch diese vollständig.

→
