# Supplementary material for: Post-surgical outcomes in transgender women: a prospective analysis of sexual function and health-related quality of life
Source: World J Urol. 2025 Sep 2;43(1):529. doi: 10.1007/s00345-025-05887-9 (PMC12405499; doi:10.1007/s00345-025-05887-9)
Supplement: Supplementary file 2 — Supplementary Material 2 [file 345_2025_5887_MOESM2_ESM.pdf]

UMM
UNIVERSITÄTSMEDIZIN
MANNHEIM

Medizinische Fakultät Mannheim
der Universität Heidelberg
Universitätsklinikum Mannheim

Universitätsmedizin Mannheim
Klinik für Urologie und Urochirurgie
Direktor: Prof. Dr. med. M. S. Michel

Name:<br>Vorname:<br>Geb.:<br>oder Etikett	Erhebungsdatum: ____________________<br>Telefonnummer: ____________________<br>E-Mail Adresse: ____________________

Sehr geehrte Patientin,
wir danken Ihnen vielmals für die Teilnahme an unserer Studie. Wir bitten Sie die nachfolgenden Fragen möglichst genau zu beantworten. Natürlich erfolgen Erfassung, Speicherung und Auswertung Ihrer Daten unter der ärztlichen Schweigepflicht und streng wissenschaftlichen Kriterien und unter Einhaltung der Datenschutzrichtlinien (genauere Details hierzu s. Patienteninformation).

1. Sind bisher schon geschlechtsangleichende oder andere Operationen im Genitalbereich durchgeführt worden (z.B. Beschneidung)?

□ ja □ nein

wenn ja: was? wann? wo? (z.B. Brustaufbau, November 2012, Klinikum xxx)

1. ____
2. ____
3. ____
4. ____
5. ____

2a. Berufliche Situation

□ in Ausbildung □ berufstätig □ arbeitssuchend □ Frührentnerin □ Rentnerin

2b. Schulabschluss

□ keiner □ Volks-/Hauptschule □ Realschule □ Gymnasium □ andere

2c. Partnerschaftsstatus (Mehrfachnennung möglich)

□ feste Partnerschaft □ verheiratet

□ verwitwet □ keine Partnerschaft □ wechselnde Sexualpartner

3. Rauchgewohnheiten:

□ Raucherin seit ____ Jahren; ____ Zigaretten/Tag

□ Ex-Raucherin seit ____ Jahren

□ Nichtraucherin

4a. Sind Sie sexuell aktiv? (Geschlechtsverkehr, Selbstbefriedigung oder Ähnliches)

□ ja □ nein

4b. Wie ist Ihre sexuelle Orientierung?

□ Frauen □ Männer □ beide □ divers/ohne Definition

5a. Ist die Orgasmusfähigkeit vorhanden?

□ ja □ nein

5b. Wie sehr sind Sie in Bezug auf Ihren Orgasmus zufrieden?

(bitte markieren Sie eine Zahl zwischen 0 [gar nicht] und 10 [voll zufrieden])

(gar nicht) **0** 1 2 3 4 5 6 7 8 9 **10** (voll zufrieden)

6a. Leiden Sie unter häufig wiederkehrenden Harnwegsinfekten?

□ ja □ nein

wenn ja wie häufig? □ 1-3 /Jahr □ 3-6 / Jahr □ mehr als 7 / Jahr

6b.Tritt bei diesen Infekten Fieber auf?

□ ja □ nein

7a. Bestehen Schmerzen im Genital- und Beckenbereich?

□ nie □ selten □ manchmal □ meistens □ immer

7b. wenn ja, wo:

____

7c. Falls ja, wie stark sind die Schmerzen im Genital – oder Beckenbereich?

Bitte markieren Sie eine Zahl zwischen 0 (keine Schmerzen) und 10 (stärkste Schmerzen).

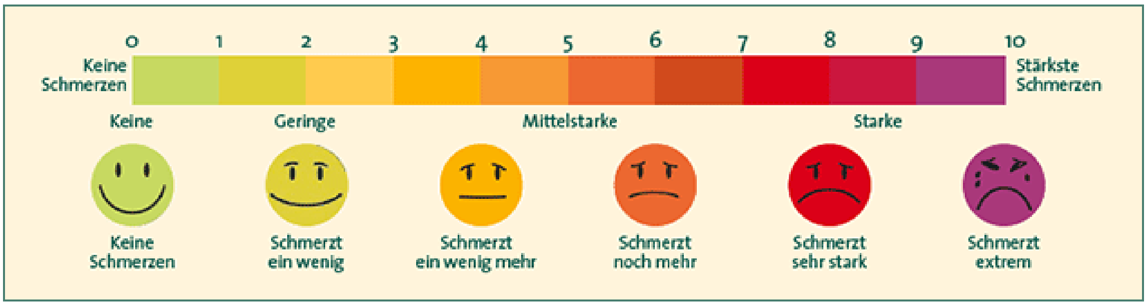

Es folgen **2 Fragebögen** zu Beschwerden beim Wasserlassen (ICIQ-MLUTS) und zum allgemeinen Gesundheitszustand (SF12). Bitte beantworten Sie auch diese vollständig.

→
